# The hope and the promise of the UN Resolution on non-communicable diseases

**DOI:** 10.1186/1744-8603-6-15

**Published:** 2010-09-09

**Authors:** George Alleyne, David Stuckler, Ala Alwan

**Affiliations:** 1 Pan American Health Organization, 525 23rd Street, N.W., Washington D.C. 20037, USA; 2 Department of Sociology, Oxford University, Manor Road, Oxford OX1 3UQ, UK; 3 London School of Hygiene & Tropical Medicine, Department of Public Health Policy, Keppel Street, London WC1E 7HT, UK; 4 World Health Organization, Assistant Director-General, Noncommunicable Diseases and Mental Health, Avenue Appia 20, 1211 Geneva 27, Switzerland

## The hope and the promise of the UN Resolution on non-communicable diseases

On May 13, 2010, the United Nations General Assembly passed resolution 265, 'Prevention and control of non-communicable diseases'[1], a major political statement calling for Heads of State to address NCDs in a 'High Level' plenary meeting scheduled for September 2011. Out of this meeting, and its associated "outcome document", will come a series of programmatic steps by all UN members. We cannot understate the potential of this UN resolution to make chronic non-communicable diseases (NCDs) a global priority among international leaders. While in the past there have been numerous resolutions in the World Health Organization World Health Assembly for greater action on NCDs, this UN resolution has special significance, as it comes with the hope to achieve multisectoral commitment and promise to deliver change. However, its overall effectiveness will depend on the ability of the international community to take advantage of this powerful political opportunity to institutionalize NCD prevention and control into policies and programmes within the broader development agenda. In this editorial we describe the kinds of outcomes that are possible and needed, and outline strategies for generating global interest as part of a social movement so to ensure commitment by Heads of State.

### Significance of the UN Resolution on Prevention and Control of Non-Communicable Disease

First, it is important to describe the genesis of the UN Resolution, because it reflects an unprecedented level of support for action on NCDs from countries of all regions. The driving force behind the resolution is the countries of the Caribbean Community (CARICOM), which, in collaboration with other countries, drafted the resolution. Their work built on two recent, important events: first, the Doha Declaration, adopted at the Ministerial Meeting on NCDs and Development, organized by the United Nations Department of Economic and Social Affairs and WHO in Doha, Qatar, in May 2009; and second, the subsequent discussions during the High-Level segment of the United Nations Social and Economic Council in July 2009, which recognized that NCDs in developing countries pose a major threat to development and called for urgent action to implement the World Health Organization Action Plan for the Global Strategy for the Prevention and Control of Non-communicable Diseases. The UN Resolution is co-sponsored by 78 countries, as well as Cameroon on behalf of the African States, including support from all geographic regions as well as representatives of the G-8 and 16 leading foreign aid donor countries.

Second, in re-framing the global discussion about NCDs, which often mistakenly focuses on 'blaming the individual' for unhealthy choices, the UN Resolution emphasises the underlying social and environmental drivers of NCDs, and their implications for poverty. As the Resolution notes, "the conditions in which people live...influence their health... and quality of life and that the most prominent non-communicable diseases are linked to common risk factors...[that] have economic, social, gender, political, behavioural and environmental determinants, and in this regard stressing the need for a multisectoral response to combat non-communicable diseases" [1]. Further, the Resolution records the "threat [NCDs] pose to the economies of Member States, leading to increasing inequalities between countries and populations, thereby threatening the achievement of the internationally agreed development goals, including the Millennium Development Goals." This framing places NCDs as a part of the UN Social Development agenda, creating space for its inclusion in the Millennium Development Goals and the post-Millennium Development Goal era after 2015.

Third, the Resolution also places chronic diseases at the centre of other development and health initiatives, including the need to strengthen health systems, the focus on prevention and control of disease, and importance of whole-of-government approaches to public health. It also builds on the work undertaken by WHO, such as the Action Plan for the Global Strategy for the Prevention and Control of Non-communicable Diseases, the World Health Organization Framework Convention on Tobacco Control, and the Global Strategy on Diet, Physical Activity and Health, and the recently endorsed Global Strategy to Reduce Harmful Use of Alcohol, specifically "recognizing the leading role of the World Health Organization."

### Three Main Policy Opportunities

Three entry points are created by the Resolution: 1) there will be a High-level Meeting of the United Nations General Assembly in September 2011, with participation of Heads of State and Government; 2) Member States are invited to speak of the socio-economic impact of NCDs and the developmental challenges they face at the MDG Summit in September 2010; and 3) the UN Secretary General will submit a Report on the global status of the NCDs to the United Nations General Assembly at its sixty-fifth session which begins in September 2010.

What can realistically be achieved as a result of these meetings and reports? Some clues are from the outcomes of past Resolutions. For example, in 2001, the HIV community held a UN high-level plenary meeting[2]. It sparked political commitments by Heads of State, and paved the way for a scale-up of funding and donor resources for addressing the HIV epidemic. While the situation facing the NCD community differs, in many respects it is similar: HIV is a chronic disease; the global communitys response to HIV was similarly belated; and, as with HIV, there is a significant potential, that left unchecked, NCDs will impose a devastating health burden on people and health systems of low-income countries, much like HIV did throughout the 1980s and 1990s. Another parallel is the UN Diabetes resolution in 2006 [3], which created a World Diabetes Day, a major rallying point for the diabetes community (albeit a relatively small-scale awareness activity in comparison with the promise of the current resolution to include NCD prevention in global development initiatives). In view of these past successes, there is little doubt that a major outcome of importance to the global effort to control NCDs will occur.

### What can concerned advocates and experts do now?

To fulfill the promise of the opportunities created by the UN Resolution, there is a critical window for those concerned about rising NCDs to act. First, the attention of Heads of State and Government must be secured to promote their participation in the meeting in September 2011. Second, while Member States will decide on the final outcomes of the meeting, international development agencies, the World Bank, UN Agencies, civil society, and the private sector must provide support through a consultative process towards the outcome document. It is hoped that the outcome will include political commitment to goals and quantifiable targets that are measured by indicators which are included as an integral part of global development initiatives. Third, stakeholders must be rallied around a common vision and road map to operationalize a global response during the next decades to come.

While the specific details will emerge from the consultative process, there are some issues that we believe are essential to include in the discussions. One is that there is a need for indicators relevant to the NCDs to be included as part of global development initiatives. Supporting the long-standing calls from the developing countries that the MDGs must take into account the prevention and control of non-communicable diseases, evidence is emerging that non-communicable diseases hold back the attainment of some of the MDGs. One recent study found that a 10 per cent reduction in rate of deaths from non-communicable diseases would have a similar impact on accelerating progress towards the tuberculosis MDG target as a a decade of economic growth in low-income countries [4]. Addressing NCDs is therefore also essential to achieving existing goals.

A second issue is national level commitments to support the implementation of the WHO Action Plan of the Global Strategy for Non-communicable Diseases and adopt coherent approaches to policy development across non-health sectors to prevent and control of non-communicable diseases. This means explicitly incorporating NCDs into poverty-reduction strategies and in relevant social and economic policies. Here, it will be necessary to adopt whole-of-government approaches to reducing the common risk factors with priority reference to tobacco, salt intake, dietary fats, especially transfats through the most effective policy interventions, including national legislation, regulation and taxation.

A third issue is the importance of extending the commitment to universal access to essential medicines and technologies, including the secondary prevention of cardiovascular disease and the treatment of cancers, diabetes, and respiratory disease. Preventing and treating NCDs must be viewed as a component of the health system strengthening agenda, in view of the inevitability of the need for long term and coordinated care for the chronic diseases (whether they are infectious such as AIDS or non-infectious in origin).

Who gets assigned the task to promote the implementation of the recommendations to be included in the outcome document is an important question. The UN resolution already gives the World Health Organization primacy in this area. WHO will have an important institutional role in rallying stakeholders, coordinating on matters where joint action is needed, providing technical support, and showing accountability. In this respect, it is crucial that mechanisms are established to provide countries and WHO with necessary resources to scale up action to prevent and control NCDs.

### Conclusion: A political platform

The UN Resolution provides a platform for the NCD community to mobilize around. Whether a movement coalesces around shared interests in response to this occasion will depend crucially on the steps taken by advocates, public health leaders, and health policy experts now. Not simply the domain of medical specialists, their control involves a wide range of disciplines and ordinary people everywhere. Relevant non-governmental organizations and other civil society actors include the recently established NCD Alliance of the International Diabetes Federation (IDF), the World Heart Federation (WHF), the International Union against Cancer (UICC) and the International Union against Tuberculosis and Lung Disease. As the knowledge and technical capacity exist to prevent and control NCDs, the central challenge now facing the NCD movement is political. The established need for action on the fiscal determinants of NCDs means that opposition from vested interests will be expected. For example, in the case of taxation, one opponent is the tobacco industry, which will continue its aggressive marketing and lobbying practices. One thing is clear: change will occur as a result of the UN Resolution. How much this change will amplify successive efforts to act on the underlying societal causes of NCDs is the principal battle to be fought.

## Competing interests

The authors declare that they have no competing interests.

## Authors' contributions

GA, DS, and AA contributed to the writing of the manuscript. All authors have read and approved the final manuscript.
